# Spinal Endermic Medication

**Published:** 1837

**Authors:** G. J. Cowles

**Affiliations:** Florence, Ala.


### Art. III—Spinal Endermic Medication.

*Cases illustrating the efficacy of medicinal applications to the region of the Spine*, by Dr. G. J. Cowles, of Florence, Ala.

As the spinal origin of diseases has but lately fixed the attention of physicians, much in relation to them, no doubt, remains to be ascertained. This will perhaps justify the publication of the following notes of cases, which have within the last two or three years fallen under my observation. I regret that they are not more precise and perfect; but defective as they may be, they must, I think, be admitted to establish one fact—the certainty with which complaints exhibiting a great diversity of symptoms, but agreeing in the presence of spinal tenderness on pressure, may be relieved and even cured, by means addressed chiefly to the region of the spine.

Case 1.—In February, 1837, I was called to visit Miss C. aged 19 years, who had been in an irregular state of health, with more or less dysmenorrhœa, for the two preceding years; and was then seized with slight hæmoptysis, which, however, did not return. For several weeks afterwards, she was affected with a mild fever of a remitting type, attended with dyspeptic symptoms, loss of appetite, debility, torpid bowels, wandering thoracic pains, fits of drowsiness in the day, and much restlessness at night; with a pulse from 100 to 120 in a minute, and often irregular. No treatment had relieved her; when on the middle of March I made an examination of the spine. It was tender throughout its whole extent. Hard pressure over the junction of the cervical and dorsal vertebræ, produced pain in the head, chest and arms, with diminution of the action of the heart, followed by a burning sensation in the external parts just named. The same kind of pressure on the dorsal vertebræ, excited pain in the abdomen: on the lumbar, pain in the lower limbs. The treatment up to this time, had consisted of mild mercurials and antimonials, cathartics and low diet; but dry cupping was now made the leading prescription. After the application, for three successive days, of cups over the most tender vertebræ, blood was drawn, and counter irritation to the back then excited, for several days longer. Very little medicine was directed. She began to mend almost as soon as the new treatment was adopted. In about a month, from imprudence, she suffered a slight relapse, but was immediately relieved by the same treatment, and since that time has had no return of her complaints.

Case II.—Oct. 5, 1836, a change of weather from mild and dry to cold and wet, had just taken place, when I was called to see Miss R. aged 12 years. Her attack was sudden, and characterized by great pain in the back of her head, in her face, eyes, and arms, with hemorrhage from her gums; occasionally there was likewise pain in the abdomen, with soreness on pressure; nausea, flatulence and costiveness were also present; her urine was scanty and red; her skin warm and dry, except that of her hands and feet, which felt cool to others, but seemed to her to be the seat of a burning sensation; her pulse was 112, contracted, and sometimes irregular; she was thirsty, and her tongue was covered with a thick fur. I directed emetics, cathartics, abdominal fomentations, and blisters to the extremities, with but little advantage, when I was led to examine the spine. From the occiput, including indeed that part, down to the middle of the back, I found great tenderness under percussion and pressure; with decided increase of suffering, in the parts supplied with nerves from this portion of the spinal cord, followed by a burning sensation in them.

Pressure on the fourth cervical vertebra, produced excruciating pain in the eyes and face. I applied a blister over the affected portions of the spine, and dressed the inflamed surface with emollient poultices, to which a small quantity of tartar emetic was added. Aperients and gentle anodynes, were the only medicines used in connexion with this local treatment, and she recovered immediately.

Case III.—In the latter part of September, 1836, Mr. S. aged 40 years, a high liver, of a bilious temperament, and an athletic frame, was attacked suddenly, with pain in the head, neck, shoulders, back, and left hip and leg. Was soon unable to sit up, or even to turn himself in bed. I did not see him till he had been ill 16 or 18 days; when I found him greatly reduced; and in addition to the symptoms enumerated, he had a full and hard pulse, beating 100 in a minute, with a white tongue, irritable stomach, irrascible mind, and a hæmorrhoidal affection. The febrile symptoms generally remitted for a part of the day, so that some degree of diaphoressis appeared—now and then even a profuse sweat. On examining the spine, I found, that from the occiput to the sacrum inclusive, there was great soreness on pressure, with very decided increase of pain in the distant parts, according to the portion of the spine on which the pressure was made. Pressure on some vertebræ produced, however, only numbness, or that feeling in one set of muscles, and pain in another. Pressure, also excited nausea, and depression, and irregularity of the pulse. The treatment usual in such cases, had been pursued without effect. Cupping was resorted to daily, and repeated for eight successive days; with calomel and opium at night, and saline cathartics in the morning. Blisters and tartar emetic ointment, were subsequently employed. He immediately began to mend, and in three weeks was nearly well. Not long afterwards, however, from some great indiscretion, he relapsed, and became as bad as ever, but was relieved by the same treatment. It is worthy of remark, that sometime before this attack, this gentleman was afflicted with an obstinate intermittent fever, which suddenly ceased, and was succeeded by diarrhœa, which after a time, as suddenly terminated, and in 24 hours afterwards, was followed by the rheumatic or neuralgic affection, which has just been described. Each change in the type of the disease, was preceded by a change of weather. He resides in a malarious district. He has not had any return of his disease, but there remains a slight paralysis of some of the muscles of his neck.

Case IV.—A negro, 40 years of age, subject to attacks of rheumatic pains, but otherwise healthy, was suddenly and violently attacked in the month of March, 1837, with severe pain in the head, right shoulder and arm, right hip and leg, and in the muscles of the chest and abdomen. He had also, a constant cough, dry skin, and hard, full, bounding pulse. Pressure along the spine, showed every vertebra to be tender, and greatly increased the pain of the parts just enumerated; augmenting the cough, and depressing the pulse. Ordered dry cupping, warm water for drink, gruel for food, and no medicine. He recovered speedily.

Case V.—Another negro, aged 44, was seized with excruciating pain in the head, neck, breast, arms and back, with palpitations of the heart, flatulence, and other gastric disturbances. He had been subject to such attacks, and they had always proved obstinate, under the plans of treatment which had been adopted. The lower part of the occiput and the vertebræ of the neck and the upper part of the back, were sore to the touch, and pressure over them augmented the pain in the muscles and joints, excited nausea, aggravated the cough, depressed the pulse and caused it to intermit. Dry cups were applied twice over the tender vertebræ, and one dose of cathartic medicine ordered, when the patient speedily recovered. Sometime afterwards, in April, 1835, he experienced a similar attack, and was treated successfully in the same way, since which he has had no return.

Case VI.—An athletic and generally healthy slave, just before I was called to see him, had suffered the extraction of two of his upper molares, on account of a horrible pain in his jaw, side of his face and ear, for which a great variety of local and general remedies had been tried, without relief. Upon inquiry I learned, that previous to the coming on of this pain, he had labored under an attack of rheumatism, which abated suddenly a few hours before the accession of the odontalgia. On examination, the whole occiput and cervical spine were found exceedingly tender; and pressure and percussion greatly augmented the pains in the face and teeth. I directed a blister to the nape of the neck, to be dressed with a poultice; and he recovered without any other treatment.

Case VII.—Mr. R. 24 years old, residing in a miasmatic region, had been subject to irregular paroxysms of intermittent fever, alternating with pain in one side of his face, upper jaw, and teeth; all of which were *partially* relieved by the sulphate of quinine. I advised a continuation of that medicine, with a blister to the back of the neck, and he was cured in a few days. In a subsequent attack, a sinapism was found to answer the purpose of a blister.

Case VIII.—June, 1836, I was suddenly called to visit Mrs. S. She was the mother of four children, had a delicate constitution, had for several years been in feeble health, with every now and then, a paroxysm of hysteria. Her menstruation had often been irregular, and her digestion depraved. I found her affected with severe spasmodic respiration, gastrodynia, and eructations of flatus to an incredible degree. On examination, all her vertebræ were tender to the touch, and the slightest pressure increased the hysterical difficulty of respiration. A sinapism over the spine produced immediate relief. She has had two or three attacks of the same kind since the one here described, and was relieved by the same application. About the time of the last attack, a blister was applied; since which her health has greatly improved. It should be stated, however, that she has latterly been more circumspect in her diet and regimen; to which no doubt, her improved health should be in part ascribed.

Case IX.—Mrs. K. aged 22 years, the mother of two children, was of a tuberculous diathesis, and exceedingly dyspeptic. Her menstrual functions were often irregular, and she had occasional slight attacks of hysteria. For three years, she had employed a variety of means without relief. On pressure, I found the lower dorsal and upper lumbar, also the cervical vertebræ tender. Dry cupping repeated twice, afforded very great relief, which was carried still further by the application, at different times of sinapisms. For twelve months she had enjoyed excellent health.

Case X.—Mr. R. of Pennsylvania, 30 years old, of a bilious temperament, athletic and generally healthy, had been much exposed in examining wild lands in the state of Mississippi, where, during the month of June, 1836, he was attacked with rheumatism in his head, shoulders and arms. Having got something better, about the middle of August he was siezed with an affection of the chest, the symptoms of which, from his narrative of them, answered well to those of an anginæ pectoris. He was then in Pontotoc, Miss. where he was treated with some success by a physician. Having come to Florence, I was desired, on the first of November, to see him. He had then, a dry and incessant cough, which had afflicted him for several weeks, with alarming palpitations of the heart; great exhaustion followed every attempt at exercise; there were fixed pains in the right shoulder and same side of the chest; and a burning sensation in the hand and foot of that side; he also labored under loss of appetite and flatulence; his pulse averaged 100 in a minute, and was soft and feeble. His skin was generally dry. He believed himself in the advanced stages of pulmonary consumption.

On examining his spine, I found great tenderness in the three lower cervical and four upper dorsal vertebræ. Cups were immediately applied, and repeated daily for a week. An emetic was administered, followed by cathartics of calomel, and minute doses of tartar emetic. An ointment with the last named medicine was also applied, so as to irritate the skin over the spine. At the end of ten days, he was so much relieved as to set off for Louisville, although the weather was cold for the season. Before he departed, I prescribed pills of rhubarb, soap and aloes as an aperient, ordered a continuance of the tartar ointment for a week longer; and directed a return to the cupping if he should get worse. From Louisville he wrote me on the 15th of December as follows:—"I arrived here in twelve days from Florence; was worse till I reached Nashville, where I rested three days; I made use of the ointment as directed, and also applied a blister. I am yet taking the pills. I feel greatly better—am indeed almost well. A little hecking cough is, I believe, the only remaining symptom, and I feel confident of being entirely well in a short time."

Case XI.—A negro aged about 35, robust and healthy, except an occasional attack of rheumatism in the knee, was seized with pain in the head and chest, April, 1837, which were followed, at no distant time, with expectoration, slight chills and paroxysms of mild fever; loss of appetite attended; he became emaciated, and was much reduced in strength; indeed, the symptoms were at length those of phthisis pulmonalis. Upon inquiry I found that this attack had succeeded to an abatement of the rheumatism in his knee joint; and it seemed to me to be a translation of that disease to the chest. On examination I found the upper dorsal and lower cervical vertebræ, exceedingly tender; and observed that all the pectoral symptoms were increased by pressure over them. Cups and irritants were applied over the spinal column; no other treatment was employed, he soon began to mend, and got entirely well.

Case XII.—A negro woman aged 30, labored under symptoms of pneumonia, and was treated in the usual way for a fortnight, without effect. The spine being examined, was found tender, and all the symptoms were aggravated by the pressure. The spinal treatment was immediately adopted, and she soon recovered. June 1837.

Case XIII.—There were more than our usual number of cases of pneumonia, in the spring of the present year, (1837,) some cases were very protracted and unmanageable. These were probably complicated with, and kept up by an affection of the spine. The case just narrated appeared to have been of this kind, and the following is another. A lad had been ill six or seven days before any decided measures were adopted, and all to which resort was then had, proved ineffectual. His symptoms were those of pneumonia. An examination of his back disclosed great soreness. The spinal treatment with cathartics, was then adopted, and, becoming immediately convalescent, he was soon entirely restored. Both in this case and the last, I was unable to detect any symptom by which the disease could be distinguished from ordinary pneumonia.

August, 1837.

